# 

**Published:** 1863-04

**Authors:** 


### DENTAL REGISTER

### OF THE WEST.

EDITED BY

J. TAFT AND GEO. WATT.

APRIL,—1863.

**MONTHLY:**

**Three Dollars per Annum, in advan[illegible]**

CINCINNATI:
J. TAFT, PUBLISHER.

J. TAFT, DENTIST, 172 RACE STREET.

